# Electroacupuncture treatment ameliorates metabolic disorders in obese ZDF rats by regulating liver energy metabolism and gut microbiota

**DOI:** 10.3389/fendo.2023.1207574

**Published:** 2023-06-27

**Authors:** Lei Ding, Rufeng Teng, Yifei Zhu, Fengming Liu, Lili Wu, Lingling Qin, Xi Wu, Tonghua Liu

**Affiliations:** ^1^ Dongfang Hospital, Beijing University of Chinese Medicine, Beijing, China; ^2^ Key Laboratory of Health Cultivation of the Ministry of Education, School of Traditional Chinese Medicine, Beijing University of Chinese Medicine, Beijing, China; ^3^ Department of Science and Technology, Beijing University of Chinese Medicine, Beijing, China; ^4^ Department of Education, Dongfang Hospital, Beijing University of Chinese Medicine, Beijing, China

**Keywords:** electroacupuncture, ZDF rats, gut microbiota, gut-liver axis, TCA cycle, oxidative stress, ROS, allantoin

## Abstract

Metabolic disorders represent a major therapeutic challenge to public health worldwide due to their dramatically increasing prevalence. Acupuncture is widely used as adjuvant therapy for multiple metabolic diseases. However, detailed biological interpretation of the acupuncture stimulations is still limited. The gut and the liver are intrinsically connected and related to metabolic function. Microbial metabolites might affect the gut-liver axis through multiple mechanisms. Liver metabolomics and 16S rRNA sequencing were used to explore the specific mechanism of electroacupuncture in treating ZDF rats in this study. Electroacupuncture effectively improved glycolipid metabolism disorders of the ZDF rats. Histopathology confirmed that electroacupuncture improved diffuse hepatic steatosis and hepatocyte vacuolation, and promoted glycogen accumulation in the liver. The treatment significantly improved microbial diversity and richness and upregulated beneficial bacteria that maintain intestinal epithelial homeostasis and decreased bacteria with detrimental metabolic features on host metabolism. Liver metabolomics showed that the main effects of electroacupuncture include reducing the carbon flow and intermediate products in the TCA cycle, regulating the metabolism of various amino acids, and inhibiting hepatic glucose output and *de novo* lipogenesis. The gut-liver axis correlation analysis showed a strong correlation between the liver metabolites and the gut microbiota, especially allantoin and *Adlercreutzia*. Electroacupuncture treatment can improve abnormal energy metabolism by reducing oxidative stress, ectopic fat deposition, and altering metabolic fluxes. Our results will help us to further understand the specific mechanism of electroacupuncture in the treatment of metabolic diseases.

## 1 Introduction

The dramatically increasing prevalence of metabolic diseases, especially type 2 diabetes mellitus (T2DM) and obesity, has become a worldwide problem over the past decades (1, 2). Metabolic diseases represent a cluster of various interrelated metabolic disorders that combine non-alcoholic fatty liver disease (NAFLD), obesity, insulin resistance, dyslipidemia, etc., which greatlly increase the morbidity and mortality of cardiovascular diseases and decrease life expectancy (3, 4). And their main characteristics are linked to abnormal energy metabolism including altered metabolic fluxes and ectopic fat deposition. The sedentary lifestyles of predominants and the existence of obesogenic environments contribute to this condition (5). Information about individual phenotypes is increasingly complex (6). Besides hyperglycemia, the T2DM phenotype typically exhibits dyslipidemia, hypertension, etc., and that are constantly treated with multiple medications (7, 8), but generally with poor efficacy and side effects consequently, which imposes a huge economic burden on the healthcare system (1, 9).

Acupuncture is an alternative therapy since ancient times been a part of traditional Chinese medicine, through the acupoints on the meridian channels that has a great therapeutic effect (10). Nowadays, this traditional technique is recognized and widely used in numerous countries, benefiting from the treatment of various diseases and its immediate curative effect (11–13). Electroacupuncture has acupoint specificity and frequency specificity (11, 14). It can stimulate reflexes by electrically stimulating acupoints on the body, thereby regulating the function of certain organs and eventually modulating physiology (10, 15–17). The efficacy and safety of acupuncture in treating metabolic disorders have been demonstrated by numerous studies (18, 19), but the specific mechanism is still unclear.

The liver is responsible for vital metabolic functions through the active and coordinated manner of multiple metabolic pathways (20, 21). However, overwhelming metabolic energy substrates lead to an imbalance of energy metabolism and toxic lipid accumulation in the liver, resulting in oxidative stress, lipid peroxidation, and mitochondrial dysfunction, leading to hepatocellular metabolic dysfunction and injury, which exacerbate the progression of hepatic steatosis and that is a shared etiology of T2DM and NAFLD (22–25). The gut microbiota acts a crucial role in the maintenance of metabolism and the absorption of nutrients (26). Disordered microbiota is closely associated with multiple metabolic diseases (27). Previous studies often ignored the potential link between the modulation of hepatic carbohydrate and lipid metabolism with dysbiosis. There are intrinsic interactions in the liver-gut axis, and approximately 70% of the blood supply in the liver originates from the intestine (2, 28). Through the effect of the microbiome and its interaction with gut-liver hormonal signaling, the gut has emerged as a main trigger to NAFLD. Accumulating evidence indicates that dysbiosis generates bioactive metabolites directly control concentrations of key metabolites in the liver to affect glucose homeostasis (26, 29), or which are absorbed through enterohepatic circulation that might indirectly lead to metabolic disorders *via* increasing accumulation of toxic compounds to the liver (2).

The obese Zucker Diabetic Fatty (ZDF) rat, which carries a mutation in the leptin receptor and exhibits severe dysregulation of hepatic glycolipid metabolism, impairment of hepatic mitochondrial fat oxidation, and decreased redox homeostasis (23, 30), is a well-established model that closely mimics human adult-onset progressive metabolic syndrome, T2DM and has been extensively employed (31).

According to the theory of “resolving phlegm and removing dampness” in traditional Chinese medicine, we selected Daimai (GB26), Zhongwan (CV12), Zusanli (ST36), and Fenglong (ST40) to form Tiao-Dai-Hua-Tan Formula (TDHT). Daimai (GB26) has been shown to improve hepatic lipid metabolism in obese rats in multiple studies (32, 33). Electroacupuncture at Zhongwan (CV12) can induce secretion of endogenous beta-endorphin which reduces plasma glucose concentration in an insulin-dependent manner (34). Zusanli (ST36) and Fenglong (ST40) are the most studied acupoints and are widely used to improve metabolic disorders through a variety of mechanisms (35, 36). We combined these acupoints to enhance the therapeutic effect synergistically and explored the underlying mechanism. Previous studies have demonstrated that electroacupuncture of TDHT can lower body weight and improve glycolipid metabolism in clinical observations. We conducted a multi-organ and multi-omics study including approaches of microbiome and metabolomics by the treatment of obese ZDF rats with electroacupuncture of TDHT. We also performed joint analyzes of the metabolome, microbiome, and host phenotypes and displayed a roadmap for the integration of models of the liver with the gut microbiota to identify potential mechanistic links.

## 2 Materials and methods

### 2.1 Materials

Electronic acupuncture treatment instrument (SDZ-II Nerve and Muscle Stimulator, 10470718029, Hwato, China) and disposable sterile acupuncture needles (0.30*25 mm, 100014261836, Hwato, China) were acquired from Suzhou Medical Appliance Factory (Suzhou, China).

### 2.2 Animals and experimental design

Male Zucker Diabetic Fatty (ZDF) rats (ZDF-Leprfa/fa) and normal control rats lean ZDF rats (ZDF-Leprfa/+) aged 7 weeks (Beijing Vital River Laboratory Animal Technology Co., Ltd., China) were raised in a temperature- and humidity-controlled environment with a 12-h light/dark cycle. The rats were fed a special diet (Purina, 5008) with free access to water. All animal experiments were conducted according to *Chinese Guidelines for Laboratory Animals* (GB14925-2001 and MOST 2006a). All animal experiments were treated following *Guide for the Care and Use of Laboratory Animals*, with approval from the Experimental Animal Ethics Committee of Beijing University of Chinese Medicine (BUCM-4-2020101903-4041).

ZDF rats (fa/fa) were randomly divided into two groups (n=10 per group) according to glucose and body weight, including the diabetes mellitus control group (DM) and the electroacupuncture group (EA). Ten lean ZDF rats (fa/+) were used as the healthy normal control group (NC). During treatment, the EA group received electroacupuncture at the following acupoints: bilateral Daimai (GB26), bilateral Zusanli (ST36), bilateral Fenglong (ST40), and Zhongwan (CV12). At the same time, the NC group was only bound without electroacupuncture treatment, while DM group was bound and sham electroacupuncture with superficial needle insertion. All rats were treated every two days for 8 weeks.

### 2.3 Assays of glycolipid metabolism

During the experiment, glucose oxidase method was used to dynamically monitor the fasting blood glucose (FBG) levels and random blood glucose (RBG) levels weekly. The oral glucose tolerance test (OGTT) was performed after treatment for 54 days. All animals were fasted for 10 h with water ad libitum, and then FBG was collected. Oral glucose loading (2 g/kg body weight) was provided, and blood collection was performed at 30, 60, and 120 min. The blood glucose was tested and the area under the blood glucose-time curve (AUC) was calculated. After 8 weeks of treatment, blood was collected through the abdominal aorta and centrifuged at 3000 r, 4°C for 15 mins. Arterial serum was used for the following tests: the serum fasting insulin (FINS) level was measured using Rat insulin ELISA KIT (Solarbio, SEKR-0033, China). The serum glucose, triglyceride (TG), total cholesterol (TC), high-density lipoprotein (HDL), low-density lipoprotein (LDL) and free fatty acid (FFA) levels were assayed by automatic biochemical analyzer (Beckman Coulter, AU480, USA) after 8 weeks of treatment. The homeostasis model assessment of insulin resistance (HOMA-IR) was calculated by the serum FINS and the serum glucose.

### 2.4 Histopathological observation of the liver

Liver tissues were fixed in 4% paraformaldehyde (Solarbio, P1110, China) and were paraffin-embedded. Multiple sections (5 mm) were prepared and stained with hematoxylin and eosin (H&E) for general morphological observation. Liver glycogen was determined using a PAS kit (Solarbio, G1281, China) following the manufacturer’s instructions. Liver tissues were used to detect the liver TC (Nanjing Jiancheng, A111-1-1, China) and liver TG (Nanjing Jiancheng, A110-1-1, China) levels by using the relevant kits.

### 2.5 Assays of gut microbiota

Fecal samples were collected after 8 weeks of treatment and the gut microbiota diversity was determined by Major Bio-Pharm Technology Co. Ltd. (Shanghai, China) according to a previously described method. Briefly, the raw 16S rRNA gene sequencing reads were demultiplexed, quality-filtered by fastp version 0.20.0, and merged by FLASH version 1.2.7. Operational taxonomic units (OTUs) with a 97% similarity cutoff were clustered using UPARSE version 7.1, and chimeric sequences were identified and removed. The taxonomy of each OTU representative sequence was analyzed using the Ribosomal Database Project (RDP) Classifier version 2.2 against the 16S rRNA database (Silva v132 http://ww.arb-silva.de) with a confidence threshold of 0.7.

The intestinal flora in feces was assayed as previously described. Fecal samples were collected and snap-frozen in liquid nitrogen, followed by storage at -80°C. Genomic DNA was isolated with DNA isolation kit. The V3–V4 region of 16S rRNA genes was amplified and purified. Subsequently, the abundance and diversity of gut microbiota were analyzed using Illumina MiSeq sequencing (Major Bio-Pharm Technology, Shanghai, China) according to the standard protocol. The effective reads from all samples were grouped into operational taxonomic units (OTUs) on the basis of 97% sequence similarity. α-Diversity was estimated by the level of OTUs, and Shannon, Ace, and Chao indices. β-Diversity was assessed by computing for unweighted UniFrac and visualized by principal coordinate analysis (PCoA) and non-metric multi-dimensional scaling analysis (NMDS). The sequence data were processed and analyzed on the online Majorbio I-Sanger Cloud Platform (www.i-sanger.com).

### 2.6 Metabolomics analysis of the liver

200 mg of the liver sample was placed in a centrifuge tube after rinsing the blood with saline, then freezing in liquid nitrogen for 15 min, and frozen at -80°C. After melting, 100 mg was placed in a centrifuge tube, 900 ul (8:1, v/v) methanol aqueous solution was added, and then ground at 60 Hz for 2 min. After grinding, 120μL chloroform was added and vortexed for 1 min, followed by ice water bath ultrasonic extraction for 10 min. After centrifugation (10000 rpmX10 min), 200 ul of the supernatant was placed into the sample bottle and then incubated dry under 37°C for 90 min for an oximation reaction. And then 15 mg/mL methoxamine hydrochloride pyridine solution 80 μL was added to it. The resultant mixture was vortexed vigorously for 2 min and placed at 37°C for 90 min. 80 μL BSTFA (containing 1%TMCS) and 20 μL n-hexane were added to the mixture, vortexed vigorously for 2 min, and then derivated at 70°C for 60 min. The samples were placed at room temperature for 30 min for GC-MS metabolomics analysis. The quality control samples which were prepared by mixing aliquots of all samples to be a pooled sample were named QC group. QC samples were used to equilibrate the system before testing and evaluate the stability of the MS system during detection. When they were closely clustered together it indicates that this assay was stable and reproducible.

Metabolomics analysis was performed on mzXML files by using Agilent MassHunter qualitative analysis software (version B.07; Agilent). XCMS Online (www.xcmsonline.scripps.edu) was used for retention time Correction, feature detection, and alignment. The XCMS-data processing results were processed by using XCMS Analyzer 3.0. Structural identification of metabolites was carried out on the basis of comparison of the molecular ion mass and MS/MS segments with reliable documentation, standards, or online databases of the Human Metabolome Database (http://www.hmdb.ca) and Metlin (http://metlin.scripps.edu). And then, the multivariate statistical analyses including Principal Component Analysis (PCA), Partial Least Squares Discriminant Analysis (PLS-DA), and Orthogonal Partial Least Squares-Discriminant Analysis (OPLS-DA), were performed on pre-processed data by using SIMCA (version14.1, Umetrics) software, so as to obtain the generalized separation of all group variations and search for biomarkers based on the variable importance in projection (VIP), respectively. The parameters of the models, including R2X, R2Y, and Q2, were analyzed to evaluate the quality of the models. When R2 > 0.5, a reliable predictive model is expected, and when Q2 > 0.9, the model is considered as having excellent predictive ability. To validate the model, cross-validation was applied to the OPLS-DA model, and the reliability was rigorously confirmed by permutation testing (n = 200). Then the data were processed and analyzed on the online Oebiotech Cloud Platform (https://cloud.oebiotech.cn).

### 2.7 Statistical analysis

All quantitative data are presented as the mean ± standard error of the mean (SEM). The SPSS 20.0 software package was used for all statistical analyses. Two-way ANOVA with repeated-measures was performed for the FBG, RBG, BW, and OGTT curve data analysis. Other data were analyzed by one-way ANOVA followed by Tukey’s tests. *P < 0.05* was considered statistically significant.

## 3 Results

### 3.1 Effects of electroacupuncture on glucose homeostasis of the ZDF rats

**Figure 1:**
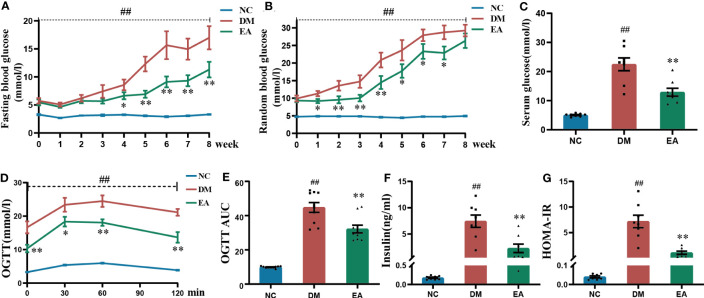
Glucose homeostasis results from ZDF rats of all groups. **(A)** Weekly fasting blood glucose. **(B)** Weekly random blood glucose. **(C)** Serum fasting glucose. **(D)** The oral glucose tolerance test. **(E)** The area under the blood glucose-time curve of OGTT. **(F)** Serum fasting insulin. **(G)** HOMA-IR. Data are shown as the means ± standard error of the mean (n=8-10, each group). DM vs. NC: ^#^ *P <* 0.05, ^##^ *P <*0.01; DM vs. EA: *P <0.05, **P < 0.01.

FBG and RBG are the most presentative and vital indicators of the original physiological state of the ZDF rats. The FBG and RBG levels of ZDF rats in different groups were measured (**Figures 1A, B**). Compared with the NC group, FBG and RBG levels were significantly higher (*P* < 0.01) in the DM group during the whole course of the experiment. After electroacupuncture treatment, the FBG levels were significantly lowered in the EA group when compared with the DM group from the 4th week until the end; The RBG levels were significantly reduced from week 1 to week 7 compared with the DM group (*P < 0.05*, 0.01). A significant decrease was also observed in the serum glucose of the EA group compared with the DM group over the experiment (*P* < 0.01, **Figure 1C**). After the treatment with electroacupuncture, the glucose of obese ZDF rats could be significantly reduced.

The results of OGTT after the 8-week treatment showed that the DM group present significantly impaired glucose tolerance when compared with the NC group (*P* < 0.01), and this trend was dramatically reversed in the EA group (*P <*0.05, 0.01, **Figure 1D**), which was also seen in the AUC of OGTT (*P* < 0.01, **Figure 1E**). Electroacupuncture significantly improved impaired glucose tolerance.

As was shown in **Figure 1F**, obese ZDF rats showed severe hyperinsulinemia when compared with those in the NC group (*P* < 0.01), and it could be reversed by electroacupuncture in the EA group when compared with the DM group (*P* < 0.01). After calculating the HOMA-IR index in each group, the results showed that the HOMA-IR index from the DM group was significantly raised when compared with that of the NC group (*P <* 0.01), whereas it was significantly decreased in the EA group (*P <* 0.01, **Figure 1G**). Therefore, electroacupuncture can significantly relieve hyperinsulinemia and insulin resistance.

### 3.2 Effects of electroacupuncture on general condition and lipid metabolism of the ZDF rats

**Figure 2:**
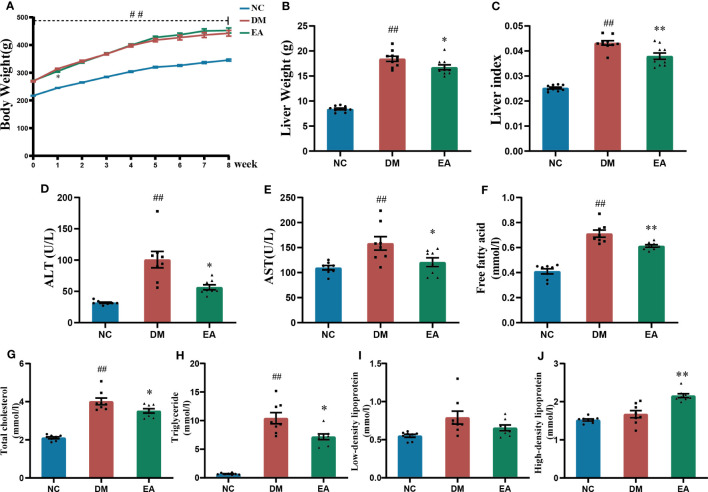
Effects of electroacupuncture on general condition and lipid metabolism of the ZDF rats. **(A)** Weekly body weight. **(B)** Liver weight. **(C)** Liver index. **(D)** Serum ALT. **(E)** Serum AST. **(F)** Serum free fatty acid. **(G)** Serum total cholesterol. **(H)** Serum triglyceride. **(I)** Serum low-density lipoprotein. **(J)** Serum high-density lipoprotein. Data are shown as the means ± standard error of the mean (n=8-10, each group). DM vs. NC: ^#^ *P <* 0.05, ^##^ *P <* 0.01; DM vs. EA: *P < 0.05, **P < 0.01.

The body weight of the obese ZDF rats was significantly increased when compared with the lean ZDF rats in the NC group (*P <* 0.01), and no significant difference between the EA group and the DM group was observed (**Figure 2A**). Obese ZDF rats developed severe NAFLD, the liver weight, liver index, and liver function including ALT and AST in the DM group were significantly higher than those in the NC group (*P <* 0.01), while these were significantly decreased in the EA group compared to the DM group (*P <*0.05, 0.01, **Figures 2B–E**). Electroacupuncture significantly relieved NAFLD in obese ZDF rats.

Dyslipidemia was observed in the DM group, with significantly higher TC, TG, and FFA levels relative to those of the NC group (*P <*0.01). After electroacupuncture treatment for 8 weeks, there were significant decreases in TC, TG, and FFA levels and a significant increase in HDL levels in the EA group (*P <*0.05, 0.01, **Figures 2F–J**). The results indicated that electroacupuncture modified dyslipidemia in obese ZDF rats.

### 3.3 Effects of electroacupuncture on histopathological characteristics, glucolipid metabolic features in the liver of the ZDF rats

**Figure 3:**
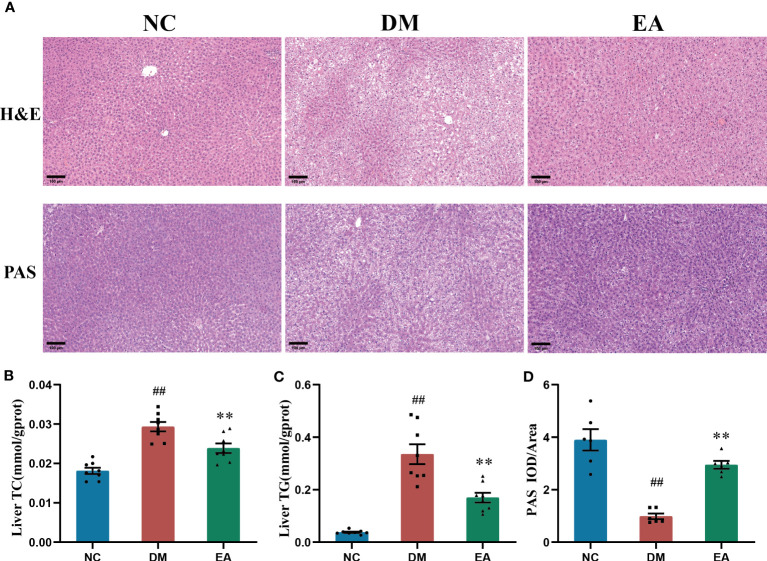
Histopathological changes in tissues of the liver, and effects of electroacupuncture on hepatic glucose and lipid metabolism in each group. **(A)** Histopathological examination(H&E) staining (200×) and Periodic Acid-Schiff (PAS) staining (200×) of liver tissues. Representative images were presented. The scale bar represents 100 μm. **(B)** Liver total cholesterol. **(C)** Liver triglyceride. **(D)** The expression intensity of glycogen by (IOD/AOI) in all groups. Data are shown as the means ± standard error of the mean (n=6-8, each group). DM vs. NC: ^#^ *P <* 0.05, ^##^ *P <* 0.01; DM vs. EA: *P < 0.05, **P <0.01.

To investigate the histopathological characteristics in the liver, HE staining and PAS staining was performed on tissue slides of the liver isolated from the ZDF rats (**Figure 3A**). Compared with the NC group, HE staining revealed marked morphological changes in the DM group, mainly manifested as diffuse vesicular steatosis, and small focal inflammatory cell infiltration, which were reversed by the electroacupuncture treatment with a significant decrease in the number and size of the lipid droplets. With the clue from the above observations in the liver, we conducted biochemical experiments on triglycerides and total cholesterol in the liver to directly examine the curative effect. The experimental results were consistent with the pathological changes, electroacupuncture treatment significantly reduced the liver TC and liver TG when compared with the DM group (*P <* 0.01, **Figures 3B**). Additionally, PAS staining of the liver sections present significantly lower glycogen deposition in the DM group than that in the NC group, whereas electroacupuncture treatment facilitated glycogen accumulation. PAS staining was quantitated by calculating the ratios of IOD to Area to evaluate the intensities of positive staining. The relative expression of glycogen in the EA group was significantly higher than that in the DM group (*P <* 0.01, **Figure 3D**). Therefore, the results above greatly proved that electroacupuncture plays an effectively protective role in hepatic pathological morphology recovery and glycolipid metabolism in the ZDF rats.

### 3.4 Effects of electroacupuncture on the overall structure changes of the gut microbiota

**Figure 4:**
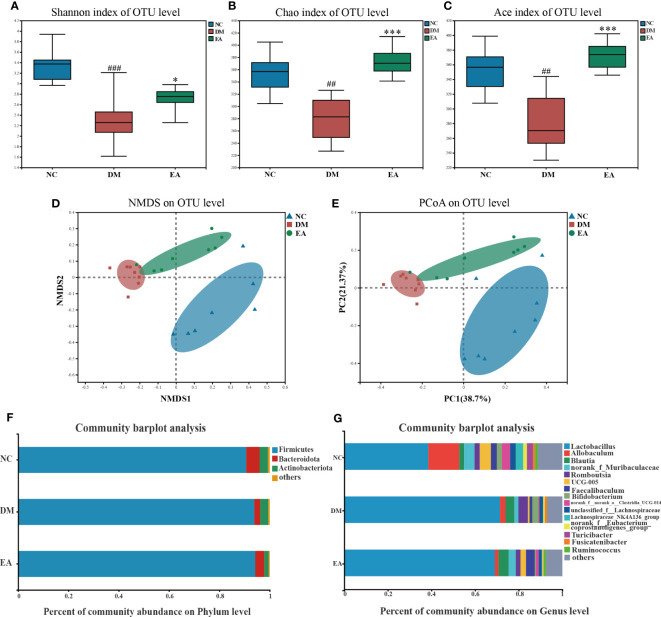
Gut microbiota structure changes from ZDF rats of all groups. **(A)** Shannon index. **(B)** Chao index. **(C)** Ace index. **(D)** NMDS of a Bray-Curtis distance matrix of the three groups at the OTU level. **(E)** PCoA of an unweighted UniFrac distance matrix of the three groups at the OTU level. **(F)** The gut microbiota composition at the phylum level. **(G)** The gut microbiota composition at the genus level. Data are shown as the means ± SD (n=8, each group). DM vs. NC: ^#^ *P <*0.05, ^##^ *P <* 0.01, ^###^ *P <* 0.001; DM vs. EA: *P < 0.05, **P < 0.01, ***P < 0.001.

The impact of electroacupuncture on microbiota composition was evaluated by using 16S rRNA gene sequencing. All effective reads were clustered into OTUs at a 97% similarity level. The α-diversity analysis has been shown in **Figures 4A–C**. Analysis of the Shannon index showed a significant drop in community diversity in the DM group (*P <* 0.001) compared with the NC group, while electroacupuncture treatment significantly increased the community diversity (*P <*0.05) of the gut microbiota. It was also observed that a significant rising trend towards the Chao and the Ace indices in the EA group which indicated a significant increase in community richness following the electroacupuncture treatment compared with the DM group (*P < *0.001). To visualize the group differences in gut microbiome composition, the β-diversity of microbial composition was calculated by using unweighted UniFrac-based principal coordinates analysis (PCoA) and non-metric multi-dimensional scaling analysis (NMDS). As shown in **Figures 4D, E**, the gut microbiota structure of the three groups can be clearly distinguished, which means that the composition of the gut microbiota in the three groups differed significantly from each other. The EA and the NC group converged to be closer to a position, suggesting that the electroacupuncture treatment made the gut microbiota structure of the EA group tend to recover to that of the NC group.

### 3.5 Effects of electroacupuncture on the gut microbiota composition of the ZDF rats

After the sequence was processed and filtered, a total of 1,042,992 high-quality valid sequences were obtained from 24 fecal samples (n = 8 per group, 43458 per sample, minimum: 38098; maximum: 51900). The number of OTUs in the EA group was increased by electroacupuncture treatment. α and β diversity analysis has confirmed that the differences in microbial communities among different groups were significant. We further searched for the signature differential gut microbiota. At the phylum level, around 99% of the total bacterial abundance was mainly composed of *Firmicutes*, *Actinobacteria*, and *Bacteroidetes*, nevertheless the remaining relatively small sequences were not classified (**Figure 4F**). The relative abundances of *Bacteroidetes* in the three groups were 5.30%, 2.27%, and 3.58%, and those of *Firmicutes* were 90.68%, 93.95%, and 94.26%. Compared with the NC group, the abundance of *Bacteroidetes* in the gut microbiome of the DM group decreased, while the abundance of *Firmicutes* increased, which are the two main communities that affect energy metabolism homeostasis. Notably, the *Firmicutes/Bacteroidetes* (F/B) ratio decreased in the DM group whereas electroacupuncture treatment reduced this difference. Electroacupuncture might affect the change in the ratio of F/B in gut microbiota.

**Figure 5:**
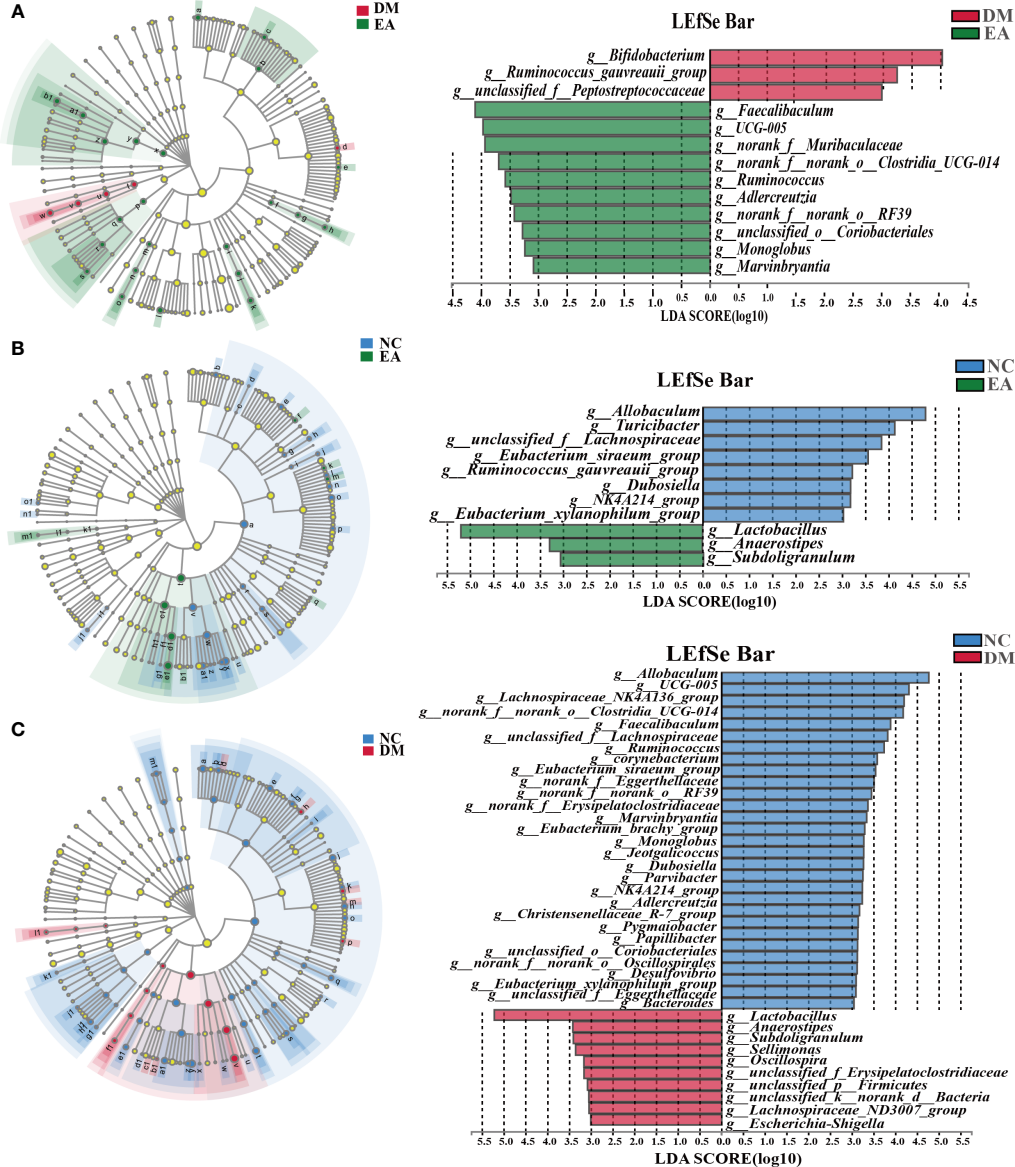
Differences in the bacterial communities at the genus level obtained by LEfSe analysis of the 16S sequences. **(A)** Cladogram of the LEfSe results between the DM and the EA groups. **(B)** Cladogram of the LEfSe results between the NC and the EA groups. **(C)** Cladogram of the LEfSe results between the NC and the DM groups. Taxonomic represent statistically and biologically consistent differences. Only the taxa with a significant logarithmic LDA threshold score of > 3 and *P <* 0.05 were shown.

The gut microbiota of each group at the genus level was shown in **Figure 4G**. LEfSe was used to distinguish the different bacterial communities between the two groups, and linear discriminant analysis (LDA) was used to estimate the bacterial communities with significant differences in abundance and the impact of the abundance of each genus on the different effects. LEfSe analysis revealed 62 discriminative features. 13 genera were significantly changed by electroacupuncture treatment when compared with the DM group (**Figure 5C**). Among these, 10 were increased: *Monoglobus*, *Marvinbryantia*, *Adlercreutzia*, *UCG-005*, *unclassified_o:Coriobacteriales*, *Faecalibaculum*, *norank_f:Muribaculaceae*, *Ruminococcus*, *norank_f:norank_o:RF39*, *norank_f:norank_o:Clostridia_UCG-014*, while 3 genera showed decreased abundances: *unclassified_f:Peptostreptococcaceae*, *Ruminococcus_gauvreauii_group*, *Bifidobacterium*. Compared with the NC group, 29 features (*Allobaculum*, *UCG-005*, *Faecalibaculum*, and others) decreased in abundance in the DM group, whereas 10 features (*Lactobacillus*, *Anaerostipes*, *Subdoligranulum*, and others) increased significantly (**Figure 5B**). The significant difference between the EA group and the NC group was only 11 features (**Figure 5A**), which means that electroacupuncture treatment partially restored the gut microbiota consistent with the NC group.

**Figure 6:**
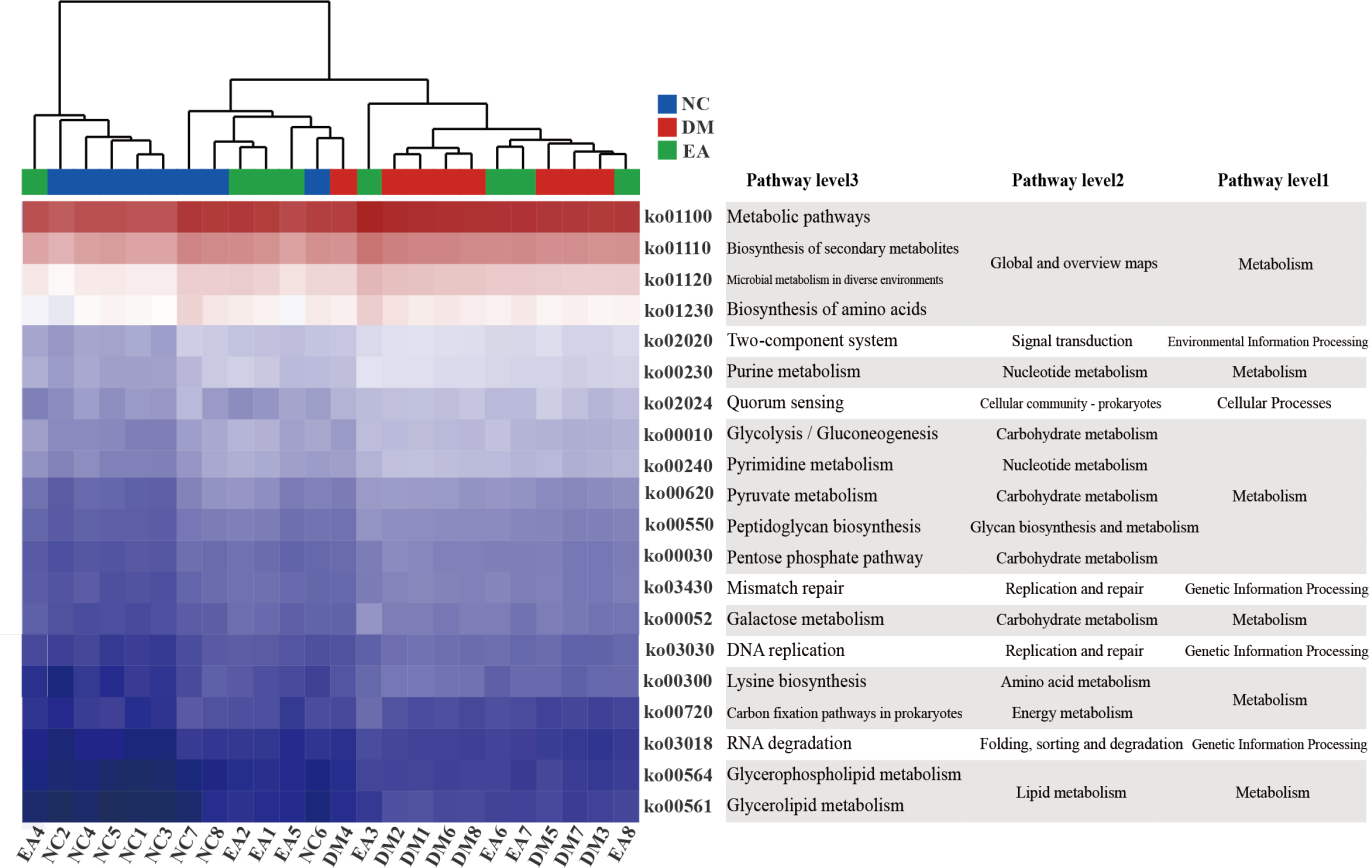
Gut microbiota functional changes. Heat map of the top 20 pathways (*P <* 0.05) with the highest relative abundance of KEGG function predicted by the microbial PICRUSt.

PICRUSt analysis and KEGG database were combined for functional prediction of the gut microbiota, and the potential metabolic pathways that might be affected were obtained. As shown in **Figure 6**, the top 20 pathways with significant differences (*P <*0.05) in gene relative abundance were mainly metabolic pathways including amino acid metabolism, carbohydrate metabolism, lipid metabolism, as well as biosynthesis of secondary metabolites and microbial metabolism in diverse environments, etc. Therefore, the metabolism of different gut microbiota and their metabolites significantly affected the metabolism of ZDF rats in different groups.

### 3.6 Effects of electroacupuncture on the liver metabolome of the ZDF rats

**Figure 7:**
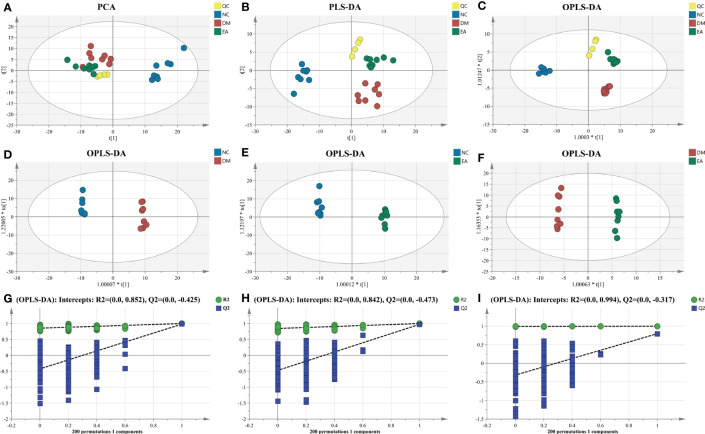
Multivariate statistical analysis of hepatic metabolic profiling of ZDF rats. **(A)** PCA score. **(B)** PLS-DA score. **(C)** Total OPLS-DA score. OPLS-DA score (**(D)** NC vs. DM; **(E)** NC vs. EA; and **(F)** DM vs. EA). Permutation test corresponding to OPLS-DA score (**(G)** NC vs. DM; **(H)** NC vs. EA; and **(I)** DM vs. EA).

First, multivariate statistical analysis was conducted to observe the overall difference between each group and the variation degree within each group. As depicted in **Figure 7A**, the PCA score plots showed little variation within each group and no obvious outlier samples were found, which examines the stability and reproducibility of the analysis. The supervised discriminant analysis methods PLS-DA and OPLS-DA were conducted to achieve better separation between different groups, so as to get a deeper understanding of the variables responsible for classification. As indicated in the PLS-DA and OPLS-DA score plots, good separation was achieved between the NC, DM, EA, and QC groups, reflecting the obvious treatment effects of electroacupuncture (**Figures 7B, C**). Additional OPLS-DA score plots of each group compared one by one are presented in **Figures 7D–F**. In these models, all the groups were clearly distinct from each other. The OPLS-DA models were further permutation tested (n = 200) to validate the robustness of the models, the results of the test confirmed that the OPLS-DA models of the metabolomics had good adaptability, high predictability, and no over-fitting (**Figures 7G–I**).

**Figure 8:**
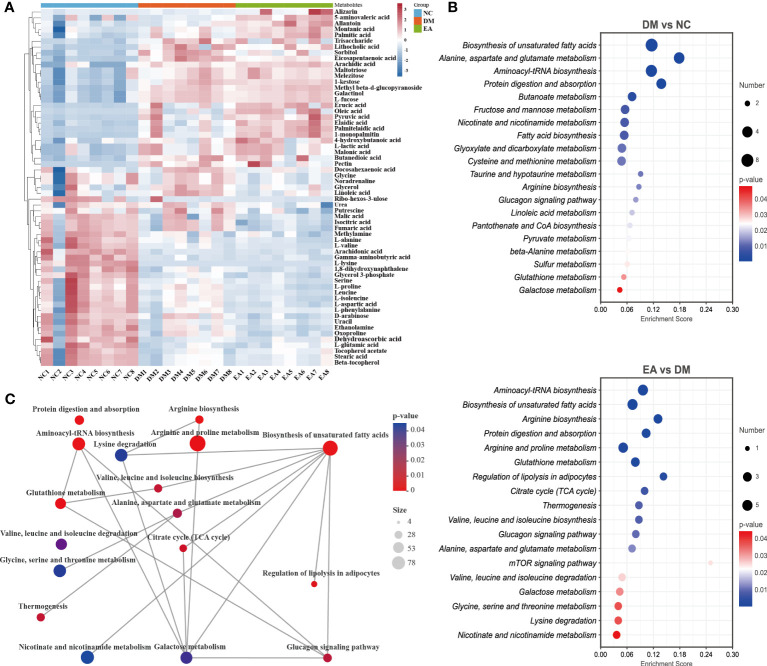
Effects of electroacupuncture on hepatic metabolic profiling of ZDF rats. **(A)** Heatmap of the identified differential metabolites. **(B)** Enrichment of differential metabolic pathways. **(C)** Network of differential metabolic pathways.

After data integration, totally 61 differential metabolites were enriched and identified (*P <*0.05, VIP > 1), mainly including fatty acids, amino acids, organic acids, etc. A total of 27 metabolites were identified as potential biomarkers in the EA group. To comprehensively analyze and visualize the relative expression levels of differential metabolites among groups and samples, a heatmap was used to observe the relative expression levels of metabolites on the basis of the analysis of Pearson correlation coefficients, so as to investigate the quality, repeatability, and similarity of the data. **Figure 8A** illustrated the differential metabolites for each sample and present the relative up-regulated (red) or down-regulated (blue) intensity of the metabolites. On the basis of the differential metabolites screened above, we studied the potential metabolic pathways after the electroacupuncture treatment, which were analyzed by KEGG metabolic pathway enrichment and depicted by bubble maps (**Figure 8B**). These metabolic characteristics mainly included: amino acid metabolism and fatty acid metabolism. Metabolomics analysis showed that electroacupuncture may improve metabolic disorders by regulating metabolites in the tricarboxylic acid (TCA) cycle, amino acid metabolisms such as arginine biosynthesis and fatty acid metabolism.

**Figure 9:**
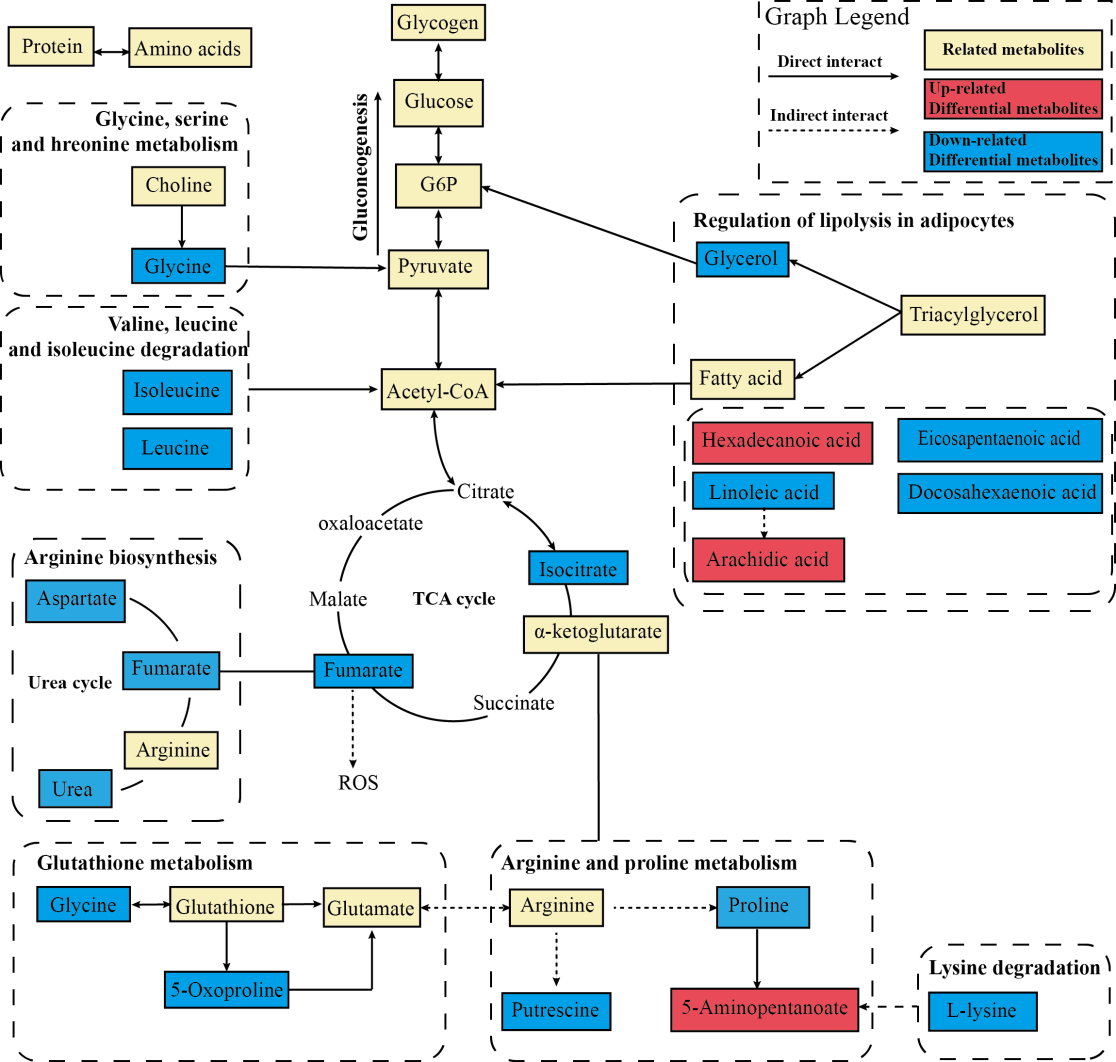
Metabolic network analysis of the identified metabolites. The profile of metabolic network analysis of the identified metabolites based on the known metabolic pathways. The solid line represents the direct interaction, while the dotted line represents the multistep reaction. The red color represents significantly increased metabolites in the EA group, while the blue represents decreased dramatically.

To further deeply investigate the relationship between metabolic pathways, the network map of metabolic pathways was drawn (**Figure 8C**). The metabolic pathways are dominated by lipid metabolism of unsaturated fatty acid biosynthesis, metabolism of various amino acids centered on aminoacyl-tRNA biosynthesis, and carbohydrate metabolism dominated by glucagon metabolic pathway, and these metabolites are connected into a network through the TCA cycle. To visualize the global metabolic network established by the potential biomarkers as indicated by the KEGG databases, this study evaluated the effects of electroacupuncture on hepatic metabolic alterations in ZDF rats in terms of the abundance of metabolic pathways (**Figure 9**). The analysis indicated that the main effects of electroacupuncture therapy include reducing the carbon flow and intermediate products in the TCA cycle, regulating the metabolism of various amino acids, and inhibition of gluconeogenesis by reducing substrates.

### 3.7 Correlation analysis between the liver differential metabolites and the gut microbiota and related parameters

**Figure 10:**
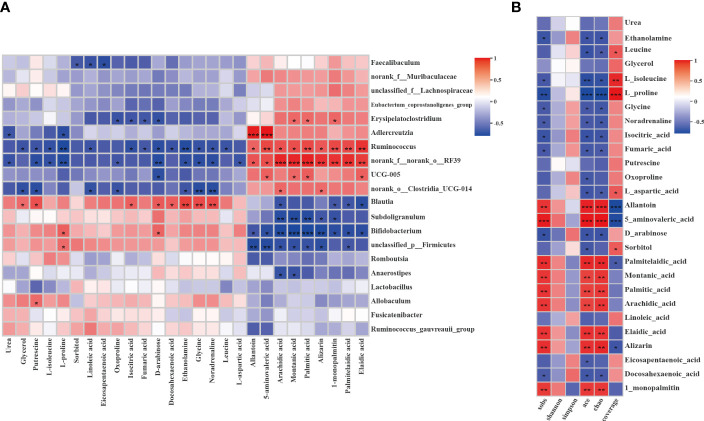
Correlation analysis between the liver differential metabolites and the gut microbiota or gut microbiota diversity and richness. **(A)** Spearman’s rank correlation between the liver differential metabolites and the gut microbiota. **(B)** Spearman’s rank correlation between the liver differential metabolites and the gut microbiota diversity and richness. The values of the correlation are shown in different colors and the color intervals are shown in the legend on the right. P value is the significance of the correlation: *P < 0.05, **P < 0.01, ***P < 0.001.

The Spearman correlation test was applied to comprehensively explore the relationships between the differential metabolites and the genera of gut microbiota (|r| > 0.5, *P <* 0.05, 0.01). As shown in **Figure 10A**, the two show a strong correlation. *Adlercreutzia* was significantly positively with allantoin and 5-aminovaleric-acid and *p:Firmicutes* was significantly negatively with them. *Ruminococcus* and *RF39* were significantly negatively with L-proline and glycine and significantly positively with fatty acids such as arachidic acid, montanic acid, palmitic acid, etc. However, *subdoligranulum*, *Bifidobacterium*, and *p_firmicutes* were significantly negatively correlated with these fatty acids. To explore the relativeness between the liver metabolites and the gut microbiota diversity and richness, we further correlated the differential metabolites and gut microbiota diversity and richness indicators using Spearman correlation analysis (**Figure 10B**). The results confirmed that metabolites and gut microbiota richness influenced each other. The Sobs, Ace and Chao indices were significantly negatively with L-proline and significantly positively with allantoin and 5-aminovaleric-acid (|r| > 0.5, *P <*0.05, 0.01). The correlations indicated that liver metabolites are tightly related to changes in gut microbiota structure.

## 4 Discussion

Metabolic disorders constitute a major public health concern. The strategies of TCM in treating metabolic disorders are mainly based on resolving phlegm and removing dampness and turbidity, and these effects are multifunctional and multitargeted (37). This kind of treatment has also been applied and studied by alternative medicine including Japan and Korea (38). Acupuncture is further integrated in alternative medicine and applied to metabolic diseases (12). In view of the data presented in this study, the obese ZDF rats (fa/fa) exhibited severe metabolic disorders including obesity, hyperglycemia, and hyperlipidemia, compared to the lean controls (fa/+), which is in agreement with previous reports obtained in this strain of rats (39). Continuous excess FFA overload progressively impairs the efficiency of mitochondrial oxidative capacity, leading to the accumulation of lipotoxic metabolites that increase reactive oxygen species (ROS) production and disrupt redox homeostasis, thereby promoting hepatic steatosis, insulin resistance, and dyslipidemia (40). After the treatment of “Tiao-Dai-Hua-Tan Formula” by electroacupuncture, the insulin resistance and impaired glucose tolerance of obese ZDF rats were significantly improved, and effectively prevent the rapid increase of FBG and RBG (**Figures 1A, B**). Meanwhile, the results of this study shows that electroacupuncture improves dyslipidemia in ZDF rats which showed significant improvement in TC, TG, and FFA levels (**Figures 2F–H**).

These biochemical evidences were further supported by histopathological findings. It was consistent with the liver pathological characteristics of ZDF rats, which were represented as marked diffuse liver steatosis and hepatocyte vacuolation in comparison with the lean ones, electroacupuncture treatment markedly reduced the area and density of adipose droplets, and significantly improved hepatic steatosis (**Figure 3A**). It also promoted hepatic glycogen accumulation (**Figure 3D**). It would be expected to redirect glucose into lipogenic pathways when insulin activation of glycogen synthase is impaired. Electroacupuncture treatment suppressed the accumulation of visceral adipose which is closely related to metabolic alterations (**Figures 3B, C**). Free cholesterol is considered a major lipotoxic molecule among hepatic lipid species that found enhanced ROS accumulation and promoted lipid accumulation and cell proliferation thereby leading to lipotoxicity which drives the progression of non-alcoholic steatohepatitis (41–43). Electroacupuncture inhibits the excessive accumulation of hepatic lipids and alleviates metabolic stress-induced liver injury in ZDF rats. This effect may be the reason for the lower liver weight and liver index, and ultimately improved liver function including ALT and AST levels (**Figures 2D, E**).

The gut microbiota has been proposed as a therapeutic target in the context of metabolic diseases. The microbial community is massively impacted by perturbations including pulses or presses that promote peristalsis of the intestine (44), which is the way electroacupuncture works to modulate gut health. Microbial diversity and functional richness are critical for maintaining stability and resilience (45). Gut microbiota may contribute to NAFLD through altered energy harvesting and reduced microbial diversity (46). Shannon, Chao, and Ace indexes, representing diversity and richness (47), were significantly decreased in the DM group (**Figures 4A–C**). However, α diversities were observably elevated after electroacupuncture treatment. Meanwhile, in terms of β diversity (**Figures 4D, E**), a measure of inter-individual variability in gut microbiome composition, the communities between the EA group and NC group were closer to each other, which indicated the communities in the two groups were similar.

At the phyla level (**Figure 4F**), *Firmicutes* and *Bacteroidetes* constitute the majority of gut microbiota in healthy individuals and are relatively stable, and their increased proportions may lead to elevated FBG and inflammatory states, particularly reducing hepatic insulin action (48). The F/B ratio was significantly increased in the DM group. However, the treatment of electroacupuncture decreased the F/B ratio. The LEfSe approach was used to identify the genus that most likely explains differences for EA therapeutic effects in different groups (**Figure 5**). We found that electroacupuncture treatment significantly increased *Faecalibaculum (* 49), *UCG-005 (* 50), *Muribaculaceae (* 51), *Ruminococcus (* 52), *Coriobacteriales (* 53), *Marvinbryantia (* 54) which was a set of short-chain fatty acid (SCFA)-producing bacteria thus maintain barrier integrity, intestinal epithelial homeostasis and inhibit inflammation. A higher abundance of *UCG-005* and *Marvinbryantia* was associated with lower HOMA-IR (55). *Coriobacteriales* can alleviate liver steatosis and inflammation and maintain normal liver function (56). *Adlercreutzia* was associated with leanness (57). And EA treatment decreased *Peptostreptococcaceae* which was associated with non-alcoholic steatohepatitis and histological severity (58). Generally, consistent with previous work showing that electroacupuncture treatment upregulated bacteria known to be associated with beneficial effects and decreased bacteria with detrimental metabolic features on host metabolism.

Metabolomic analysis of the liver was performed by GC which used suitable for fatty acids and amino acids with good selectivity and sensitivity. In this study, the metabolic alterations in the DM group mostly focused on several metabolic pathways compared to the NC group (**Figure 8B**), including fatty acid biosynthesis and amino acid metabolism. And it also includes butanoate metabolism which is essential for homeostasis at the colonic epithelium (59). The analysis results confirmed the effect of intestinal microbiota on the liver of ZDF rats. Compared with the NC group (**Figure 8A**), multiple fatty acids were significantly increased in the DM group including palmitic acid, oleic acid, linoleic acid, etc. Excess lipid accumulation and abnormal fatty acid oxidation promote lipotoxicity to drive oxidative stress *via* mitochondrial uncoupling and affect mitochondrial function. Macrovacuolar steatosis coupled with chronic abnormalities of mitochondrial function exacerbates oxidative stress leading to steatohepatitis and cirrhosis (60). Lactate accumulation was observed in the DM group, and γ-aminobutyric acid, which acts to suppress glucagon secretion when glucose is elevated (61), was significantly decreased, thus forming a vicious cycle of glycolipid metabolism disorder.

As in previous studies, TCA cycling activity and pyruvate carboxylase flux were normal in the obese ZDF rats compared to the lean ZDF rats (23), whereas our results showed significant differences in the TCA cycle between EA and DM groups (**Figure 9**). The TCA cycle constitutes an epicenter in cell metabolism in mitochondria which release ROS. Studies have confirmed that greater hepatic TCA is associated with the conversion of lactate to glycerol as a substrate for gluconeogenesis, increased mitochondrial metabolism, and glyceroneogenesis, which may contribute to a vicious cycle in the pathogenesis of NAFLD and T2DM (62). Increased hepatic TCA cycle flux rate is associated with obesity people with steatosis. This is consistent with the results of our study, in which the DM group had a significant lactate accumulation compared with the NC group, while the glycerol and TCA cycle flux decreased significantly after EA treatment (63). The resulting substrate reduction of the TCA cycle further mitigate their lipotoxic potential in other organ systems (64). And electroacupuncture reduced the products of the TCA cycle which can be co-opted in pathologic states including fumarate and isocitrate. Studies have shown that elevated levels of fumarate increase ROS signaling by binding glutathione and contribute to a progressive deterioration of β cells. Electroacupuncture treatment regulated the metabolism of various amino acids. The urea cycle, dominated by arginine metabolism, was significantly inhibited in the EA group. Correlations between a disordered urea cycle and dyslipidemia have been observed in various liver diseases. Liver steatosis led to urea cycle dysfunction and worsened fibrosis progression due to the intrahepatic accumulation of toxic ammonia (65). Proline metabolizes to uric acid in the liver, and its abnormal metabolism is associated with hyperuricemia. Electroacupuncture reduced the level of branched-chain amino acids (BCAA) including leucine and isoleucine. Chronic overload from BCAA on the liver can interfere with hepatic insulin signaling and contribute to dysfunctional TCA cycle activity, this impaired cross-talk between BCAA and hepatic TCA cycle is a contributing mechanism to NAFLD (66). Moreover, electroacupuncture treatment significantly reduced glycerol, glycogenic amino acids, and acetyl-coA, which promote hepatic gluconeogenesis through substrate push mechanisms and allosteric and thereby inhibited hepatic glucose output and *de novo* lipogenesis. Therefore, electroacupuncture treatment can improve abnormal energy metabolism and alleviate steatohepatitis by reducing oxidative stress, ectopic fat deposition, and altering metabolic fluxes.

Increasing evidence shows that the intestinal microbiota and its metabolites enter the liver through the portal system (58), which plays a crucial role in liver dysfunction. Correlation analysis was conducted to find the potential molecular biomarker between the liver metabolites and bacterial abundance and diversity. The metabolite with the highest correlation value is allantoin, which increased significantly in the EA group after electroacupuncture treatment when compared with the DM group (**Figure 10**). The nitrogen-rich heterocyclic compound allantoin is an intermediate metabolite of purine catabolism in the liver (28). Allantoin is widely recognized as an antioxidant (67) that may function in oxidative stress protection and cell death by scavenging ROS (68). The ROS accumulation, a toxic mediator, can induce oxidative damage, mitochondrial apoptosis, and inflammatory response and then drive fibrosis (60). It has been reported that stress tolerance is significantly impaired due to deficiencies in xanthine oxidation, suggesting allantoin may be involved in oxidative stress protection (68). In addition, allantoin has a hypoglycemic effect, due to its metformin-like structure, it regulates glucose levels by binding to the guanidine-like structure of the imidazoline I-2 receptor and facilitating the beta-endorphin release. And it can inhibit lipid metabolites and increase glucagon-like peptide-1 to improve β cell function. A decrease in allantoin indicates a potential increased risk of insulin resistance (69). In our study, allantoin was positively correlated with *Adlercreutzia*, *Ruminococcus*, and *RF39*. Among them, the correlation with *Adlercreutzia* was extremely significant (*P <0.001*). *Adlercreutzia* is proven to be beneficial to health and to protect the intestinal epithelial barrier through anti-inflammatory effects (70, 71). Studies have confirmed that *Adlercreutzia* may alleviate NAFLD through the involvement of bile acids in the liver-gut axis as a probiotic (72). Moreover, the increase of its abundance is associated with the decrease of blood glucose and lipid levels, the inhibition of adiposity accumulation, and the improvement of glucose tolerance (57, 73), which is consistent with our results. *Ruminococcus* is also known to be beneficial for its anti-inflammatory or pro-inflammatory effects (70). Allantoin was negatively correlated with *Bifidobacterium*, possibly due to the accumulation of lactate in the DM group. It has been confirmed that when lactate concentration accumulates, it will cause an increase in the abundance of *Bifidobacterium*, this is also consistent with our results. Therefore, electroacupuncture may play a therapeutic role by enhancing the mutual crosstalk between allantoin and probiotics, in particular *Adlercreutzia*.

Although the purpose of this study is to provide a deeper understanding of the potential interaction between the gut microbiome and liver metabolome after electroacupuncture treatment with TDHT for regulated metabolism, there were several limitations. First, this is still an extensive study on intestinal microbial changes and metabolic outcomes, further efforts are necessary to focus on the precise molecular mechanisms. Second, the mechanism of other target organs causing metabolic disorders needs to be studied jointly. Third, fecal microbiota transplantation experiments and verification of metabolites need further exploration. Finally, it should be noted that the human metabolic system is far more complicated than that of rats, the results of this study only reflected the microbial population and the metabolic changes of ZDF rats subjected to electroacupuncture, rather than those in people with metabolic disorders. The clinical studies are necessary to evaluate the relevance of these results in the future.

## 5 Conclusions

In summary, our study indicates that electroacupuncture can improve severe metabolic disorders in ZDF rats. Particularly, its action mechanism is to improve abnormal energy metabolism by reducing oxidative stress, ectopic fat deposition, and altering metabolic fluxes. All in all, the results of this study provide new evidence and insights into the role of electroacupuncture in the treatment of metabolic diseases.

## Data availability statement

The datasets presented in this study can be found in online repositories. The names of the repository/repositories and accession number(s) can be found below: PRJNA968151 (SRA) and MTBLS7809 (Metabolights).

## Ethics statement

The animal study was reviewed and approved by the Experimental Animal Ethics Committee of Beijing University of Chinese Medicine.

## Author contributions

XW and TL designed the scheme of this study. LD, RT and YZ performed the experiments and conducted data extraction. LD and RT analyzed the data and made figures. YZ and FL drafted the manuscript. LW and LQ reviewed and revised the manuscript. All authors contributed to the article and approved the submitted version.
